# A Case of Xanthogranulomatous Pyelonephritis Associated with Renoduodenal Fistula

**DOI:** 10.1155/2017/8069205

**Published:** 2017-03-23

**Authors:** R. Conor Holton-Burke, Mini Varughese

**Affiliations:** Baylor College of Medicine, One Baylor Plaza, BCM620, Houston, TX 77030, USA

## Abstract

Xanthogranulomatous pyelonephritis (XGP) is a rare form of pyelonephritis associated with repeated infection, chronic inflammation, and obstruction. Various fistulas, including those to the intestine, are a known association with XGP. Here, a 55-year-old woman with a history of multiple previous renal calculi presented with dysuria and back pain. Contrast-enhanced computed tomography (CT) revealed a soft tissue density in her renal pelvis and perirenal space consistent with XGP along with a tract connecting the upper pole of her right kidney to the second portion of the duodenum. This finding was subsequently confirmed during percutaneous nephrostomy placement, stent placement, a small bowel follow-through study, and upper endoscopy. She was diagnosed with XGP with associated renoduodenal fistula, eventually treated by open nephrectomy with fistula takedown. Histopathologic analysis was consistent with the diagnosis of XGP with no malignant or infectious cause of the fistula. XGP should be considered in the diagnosis of patients with dysuria and back pain, especially when a history of obstruction or chronic inflammation. Associated fistulas should also be considered prior to surgical management to prevent complications.

## 1. Introduction

We report the case of a 55-year-old female presenting with dysuria and costovertebral angle tenderness who was found to have right-sided xanthogranulomatous pyelonephritis (XGP) associated with renoduodenal fistula. This finding was confirmed by CT, percutaneous nephrostomy placement, stent placement, small bowel follow-through study, and upper endoscopy. Histological analysis of the small bowel biopsy revealed no malignant or infectious etiology.

## 2. Case Presentation

**Figure 1:**
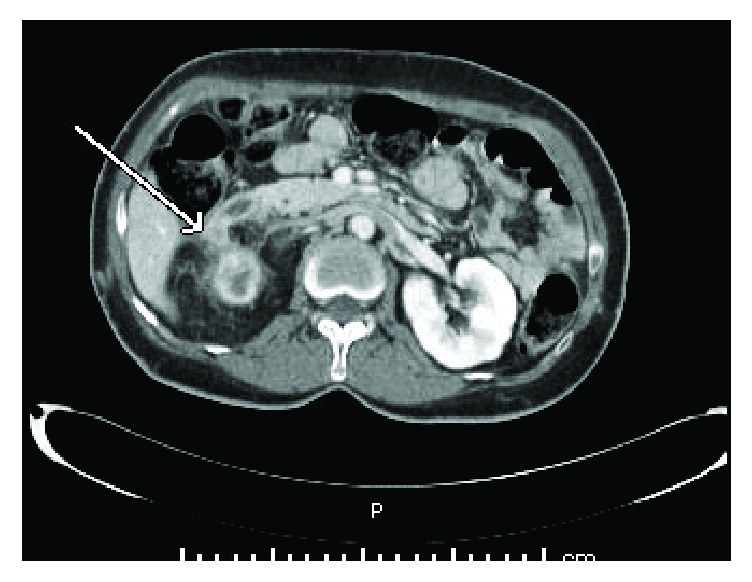
CT scan of the abdomen demonstrating a fistula between the upper pole of the right kidney and the duodenum.

A 55-year-old female with a history of recurrent nephrolithiasis was admitted with a 2-month history of progressive fatigue, weight loss, dysuria, and fevers. She had no prior history of urological instrumentation. On examination, she was found to have mild bilateral lower quadrant abdominal tenderness and pronounced costovertebral angle tenderness on the right side. Initial laboratory evaluation revealed pyuria, but no hematuria or leukocytosis. A contrast-enhanced CT of her abdomen and pelvis revealed a soft tissue density in the pelvis and perirenal space along with multiple intrarenal hypodense collections consistent with xanthogranulomatous pyelonephritis. Two small, nonobstructing calculi were identified in the collecting system and the right calyx with associated inflammatory changes. Additionally, a tract extending from the upper pole of the kidney to the second portion of the duodenum was visualized (Figure 1).

**Figure 2:**
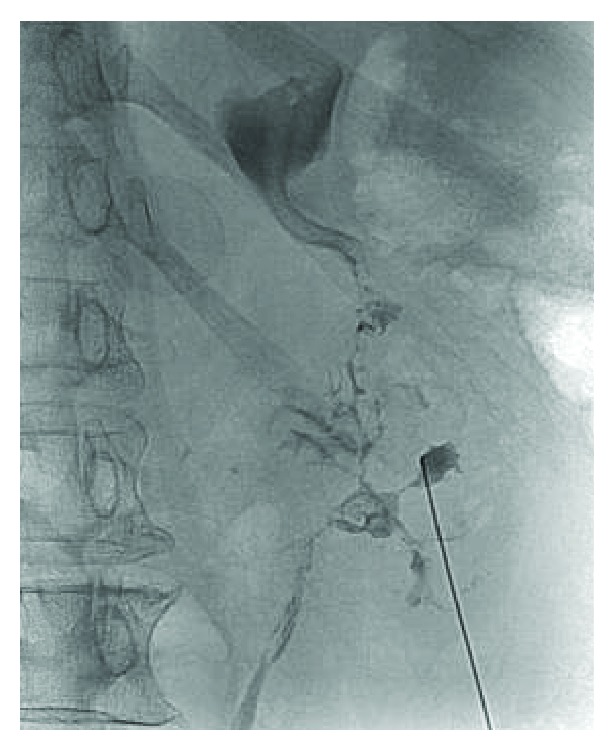
Contrast flowing from right renal calix into duodenum and stomach during attempted PCN placement.

**Figure 3:**
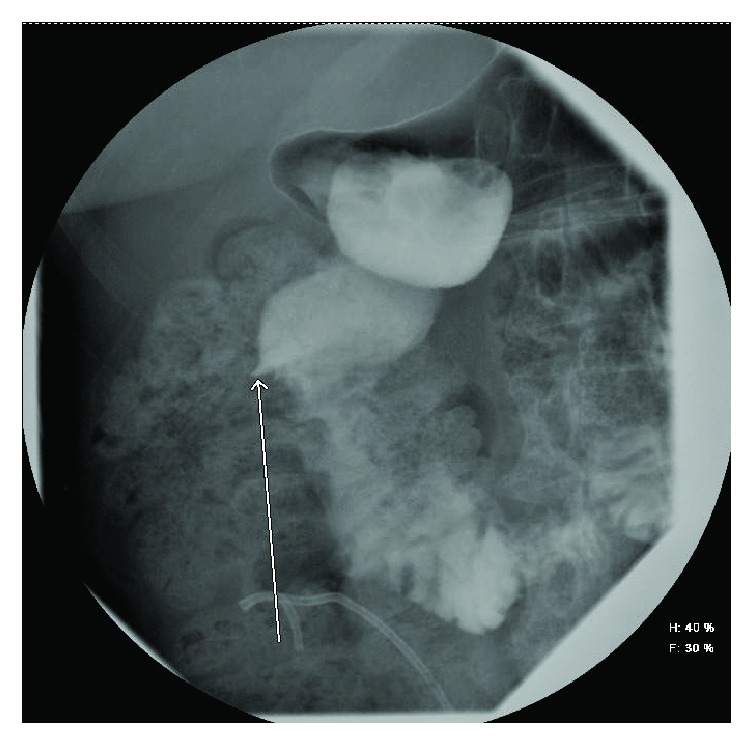
Small bowel follow-through study demonstrating contrast flowing from the second stage of the duodenum through a fistula towards the right kidney.

During subsequent percutaneous nephrostomy placement, contrast was visualized extending from the renal calyces into the patient's duodenum and stomach (Figure 2). Contrast was again visualized flowing to the small bowel and stomach during ureteral stent placement. A small bowel follow-through study confirmed the presence of a fistula extending from the second portion of the duodenum to the superior pole of the right kidney (Figure 3). Upper endoscopy was performed to rule out a malignant etiology, once again confirming the presence of a renoduodenal fistula. Biopsy of an area of nodularity near the fistula in at the D1/D2 duodenal junction revealed inflammatory changes and no evidence of malignancy.

After ureteral stent placement and eight days of treatment with IV ceftriaxone, the patient's fatigue, dysuria, and costovertebral angle tenderness resolved. A diagnosis of XGP associated with spontaneous renoduodenal fistula and was made, and the patient was discharged in stable condition on oral antibiotics. After discharge, an outpatient open nephrectomy with take-down of the renoduodenal fistula was performed. Gross findings and surgical pathology revealed acute on chronic pyelonephritis with multinucleated giant cells, consistent with XGP.

## 3. Discussion

First described by Schlagenhaufer [1], XGP is an uncommon form of pyelonephritis frequently occurring in the setting of repeated infections, chronic obstruction, and inflammation. Though the precise pathophysiology is not yet elucidated, it is thought that chronic obstruction and inflammation trigger the proliferation of lipid-laden macrophages, which leads to suppuration and renal parenchymal destruction. This theory is supported by the observation that urinary tract calculi are present in 70–79% of patients with XGP [2]. XGP accounts for approximately 1% of pyelonephritis cases reported worldwide [3].

Though rare in the general population, XGP is a relatively common variant found in surgically managed pyelonephritis. In one series, it was found to be the histopathological diagnosis in 20% of such cases [4]. Epidemiologically, it is found most commonly in women aged greater than 40 years old [4]. Diabetes mellitus, immune compromise, and abnormal lipid metabolism are also thought to be risk factors for developing this condition. Clinical presentation is nonspecific with fever, weight loss, and lower urinary tract symptoms being most common [3]. Accordingly, imaging helps with diagnosis, with both ultrasound and CT representing sensitive diagnostic modalities [5]. Though not present in our case, the “bear paw sign,” in which cysts filled with lipid-laden macrophages dilate the renal pelvis and calices, is a common radiologic finding in cases of XGR [6]. Squamous cell carcinoma of the kidney is known to mimic XGP radiographically, while XGP has been known to mimic both Wilms tumor and Renal Cell Carcinoma, so definitive diagnosis must be made histologically [7, 8]. Because XGP leads to parenchymal destruction rendering the affected kidney nonfunctional, nephrectomy is the definitive treatment [9].

Though XGP is usually limited to the affected kidney, it occasionally spreads to adjacent tissues. Malek and Elder classified the spread of the disease into the following stages [10].

*Stage I (Nephric).* Disease is confined to the renal parenchyma. 

*Stage II (Nephric and Perinephric).* Disease processes are involving both the parenchyma and the perinephric fat. 

*Stage III.* Disease is extending to the adjacent structure or retroperitoneum.

Among Stage III disease, fistula formation is known to be associated with XGP, with reports of fistulas forming to the skin, bronchi, psoas muscle, and intestine [11, 12]. These are likely due to chronic inflammation leading to adherence and subsequent perforation of renal tissue to adjacent structures, though it is also possible that initial fistula formation leads to chronic inflammation and subsequent XGP. Depending on the case series, fistulas may be found in up to 8% of cases of XGP. Among intestinal fistulas, renocolic fistulas are by far the most frequent, likely due to the abutment of the naked posterior wall of the left colon to the anterior surface of the left kidney [12]. To our knowledge, there is only one prior report of XGP associated with a renoduodenal fistula [5]. As was performed in this case, treatment for XGP with concomitant fistula formation is surgical nephrectomy with simultaneous fistula repair.

Though XGP remains a rare entity to encounter in clinical practice, it is important to be aware of the possibility, especially in patients with chronic urolithiasis. When XGP does occur, one must be cognizant of the possibility of a fistula prior to surgical management to avoid surgical complications in the patient.
